# Modelling of Behavior for Inhibition Corrosion of Bronze Using Artificial Neural Network (ANN)

**DOI:** 10.3390/e20060409

**Published:** 2018-05-26

**Authors:** D. Elusaí Millán-Ocampo, Arianna Parrales-Bahena, J. Gonzalo González-Rodríguez, Susana Silva-Martínez, Jesús Porcayo-Calderón, J. Alfredo Hernández-Pérez

**Affiliations:** 1 Posgrado en Ingeniería y Ciencias Aplicadas del Centro de Investigación en Ingeniería y Ciencias Aplicadas (CIICAp-IICBA), Universidad Autónoma del Estado de Morelos (UAEM), Cuernavaca C.P. 62209, Mexico; 2 CONACyT-Centro de Investigación en Ingeniería y Ciencias Aplicadas (CIICAp-IICBA), Universidad Autónoma del Estado de Morelos, Cuernavaca C.P. 62209, Mexico; 3 Centro de Investigación en Ingeniería y Ciencias Aplicadas (CIICAp-IICBA), Universidad Autónoma del Estado de Morelos, Cuernavaca C.P. 62209, Mexico

**Keywords:** corrosion, bronze, ketoconazole, Electrochemical Impedance Spectroscopy (EIS), Artificial Neural Network (ANN)

## Abstract

In this work, three models based on Artificial Neural Network (ANN) were developed to describe the behavior for the inhibition corrosion of bronze in 3.5% NaCl + 0.1 M Na_2_SO_4_, using the experimental data of Electrochemical Impedance Spectroscopy (EIS). The database was divided into training, validation, and test sets randomly. The parameters process used as the inputs of the ANN models were frequency, temperature, and inhibitor concentration. The outputs for each ANN model and the components in the EIS spectrum (Z_re_, Z_im_, and Z_mod_) were predicted. The transfer functions used for the learning process were the hyperbolic tangent sigmoid in the hidden layer and linear in the output layer, while the Levenberg–Marquardt algorithm was applied to determine the optimum values of the weights and biases. The statistical analysis of the results revealed that ANN models for Z_re_, Z_im_, and Z_mod_ can successfully predict the inhibition corrosion behavior of bronze in different conditions, where what was considered included variability in temperature, frequency, and inhibitor concentration. In addition, these three input parameters were keys to describe the behavior according to a sensitivity analysis.

## 1. Introduction

Copper and alloys use are very common in the industry due to their multiple properties such as high electrical and thermal conductivities, mechanical workability, and corrosion resistance. One of the principal copper alloys is represented by bronze that forms a protective layer when it is exposed to the environment. However, the environmental pollutants are more aggressive and it is necessary to increase protection [1,2].

In order to decrease the effects of corrosion, organic inhibitors offer a good alternative. They are a practical and economically feasible strategy to attenuate the economic impact that is generated by the structural damage of material loss, and can contribute to the preservation of equipment and structures in optimal conditions [3,4]. The characteristics of organic compounds with the potential to protect metals from corrosion contain heteroatoms such as nitrogen, oxygen, sulfur, phosphorous or *π* bonds, which act as active centers to adsorb on the metal surface [5,6,7,8].

Recently, pharmaceutical drugs have attracted special attention given that they share a similar characteristic in their chemical structure. The azole group and derivatives have been used widely to protect copper and alloys. According to Antonijevic and Petrovic (2015) [9], the copper atom presents vacant *d* orbitals that form bonds with heteroatoms that donate electrons or generate an interaction with rings containing conjugated bonds, *π* electrons. The complex forms a protective film on the metallic surface that blocks aggressive ions [10,11,12].

Based on previous research, the electrochemical behavior of bronze was investigated in a corrosive electrolyte (3.5% NaCl + 0.1 M Na_2_SO_4_) in the presence and absence of ketoconazole as a corrosion inhibitor at 25, 40, and 60 °C. The electrochemical evidence exhibited that ketoconazole inhibits the corrosion of the bronze, forming a protective layer with its conjugated bonds and nitrogen atoms that decrease both the charge transfer and the diffusion of aggressive species towards the metal surface. Hence, the ketoconazole acts as an adequate mixed type corrosion inhibitor [11].

Nowadays, it is necessary to develop other alternatives to better understand corrosion phenomena, reduce time, the number of experiments, as well as control the process. ANN models represent a good option to describe corrosion behavior [12,13]. This kind of a model is based on the biological functions of the brain where connections of neurons form a network. The prediction performance depends on a learning stage and corresponds to the correlation of the inputs and outputs of the model [14,15]. Some works have already demonstrated the efficiency of these models in corrosion systems using different conditions. For example, in the prediction of corrosion inhibition in pipeline steel [16,17,18], the resistance of dental metallic [19], to determine inhibitor efficiency applied in aluminium [20], and others [21,22,23,24,25,26,27,28].

Therefore, the present work aims to develop three ANN models based on the experimental data of EIS. These models are used to describe the behavior of the corrosion process of bronze with ketoconazole as an inhibitor and determine the different effects of the critical parameters, such as concentration, temperature, and frequency of the inhibitor in the EIS spectrum. Consequently, these models will be able to determine the corrosion in real time, decreasing the time and cost of experimentation for other conditions of the same system of bronze/electrolyte.

## 2. Experimental

The experimental database was prepared with the results obtained in EIS at 24 h, the bronze was exposed to the corrosive electrolyte (3.5% NaCl + 0.1 M Na_2_SO_4_) at 25, 40, and 60 °C with inhibitor concentrations of 0, 5, 10, 25, 50, and 100 ppm. All electrochemical measurements were performed in a typical three-compartment glass cell using a calomel electrode and graphite as a reference and counter electrode, respectively [11].

EIS is widely used for the characterization of film inhibitor protection on the metal surface and to understand the physicochemical properties of the system mechanism reactions. This technique consists of applying low voltage as a perturbation signal allowing the measurement of the current response at a different frequency to develop the EIS diagram [27,29,30]. EIS spectrum classification is a Nyquist and Bode diagram; the first one contains Z_re_ (Ω·cm^2^) and Z_im_ (Ω·cm^2^); the second Z_mod_ respect to frequency (Hz).

## 3. Artificial Neural Network Methodology

### 3.1. Database Preparation

The database was composed by three inputs at the ANN: Temperature (°C), inhibitor concentration (ppm), and frequency (Hz). The outputs for each model were represented by Z_re_ (Ω·cm^2^), Z_im_ (Ω·cm^2^) and Z_mod_ (Ω·cm^2^). Table 1 shows the interval work for each input and output for the ANN model.

### 3.2. Normalization Input Data

A satisfactory normalization is one of the most important aspects of the training process, which represents a direct influence on the model and offers benefits such as suitable results and a considerable decrease in calculation time [31]; because of that, all samples were normalized in the range of 0 to 1. The input database $x_{i,Real}$ (from the training, validation, and test sets) were scaled to a new normalized value $x_{i,Norm}$ using Equation (1) [32,33]:

(1) $$x_{i,Norm}=0.8\left(\frac{x_{i,Real}-x_{\min}}{x_{\max}-x_{\min}}\right)+0.1$$

### 3.3. Development of ANN Models

Matlab^®^ software (R2015b, Mathworks^®^, Natick, MA, USA) was used for the development of the three models, evaluating different combinations of activation functions and the number of neurons was increased until the best correlation between input and output variables was achieved. The training process was purposed to minimize the prediction error of the ANN through the different connections between weights and biases; it was possible using the hyperbolic tangent sigmoid transfer function in the hidden layer and linear transfer function in the output layer.

**Figure 1:**
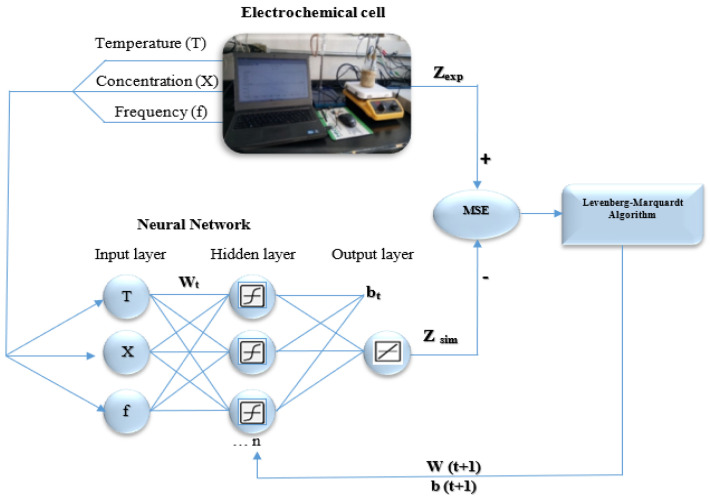
Numerical method for the artificial neural network (ANN) learning process used to predict Z_re_, Z_im_ and Z_mod_.

**Figure 2:**
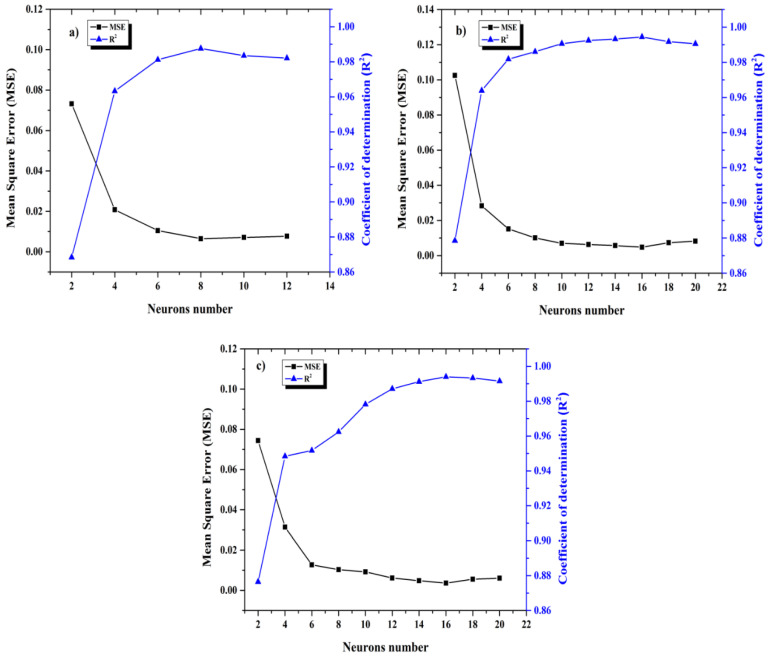
Mean Square Error (*MSE*) and Coefficient of determination (*R*^2^) in function of neurons number for each ANN model (**a**) Z_re_; (**b**) Z_im_ and (**c**) Z_mod_.

The Levenberg–Marquardt algorithm was used to determine the optimum values of the weights and biases using two parameters of the Mean Square Error (*MSE*) and the Coefficient of determination (*R*^2^). The database was randomly divided into training (60%), test subsets (20%), and validation (20%). Remarking that the last percentage corresponds to new data meaning than the validation values were not used during training. In order to obtain a good performance model and the optimum architecture, it was necessary to decrease differences between experimental and simulated values, increasing the number of neurons in the hidden layer gradually and determining *MSE* and *R*^2^ at the same time to find the minimum value for *MSE* and maximum for *R*^2^, (Figure 1); when the *MSE* increased, the training was stopped because at this moment its generate overfitting in ANN and the performance associated to *R*^2^ value could not improve, such as in Figure 2, where the plot represents the *R*^2^ and *MSE* function of the number of neurons in the hidden layer for each ANN model.

### 3.4. Statistical Analysis of Experimental and Predicted Data

The *MSE* parameter is commonly used to quantify the differences between the experimental and simulated values of the developed models. The *R*^2^ presents the strength of the linear proportion of variability in a dataset, and is the most often seen number between 0 and 1, and *R*^2^ near to 1 indicates that a regression line fits that data well [32]. Furthermore, the intercept-slope test (slope = 1 and intercept = 0) was achieved to validate the linearity and exactitude model [34].

The results obtained with ANN models were compared with the experimental data. The statistical test parameters are describing in the following equations:

(2) $$MSE=\frac{1}{N}\overset{n}{\underset{i=1}{\sum }}{\left(P_{sim\left(i\right)}-P_{\exp\left(i\right)}\right)}^{2}$$

(3) $$R^{2}=1-\frac{\sum_{i=1}^{n}{\left(P_{exp\left(i\right)}-P_{sim\left(i\right)}\right)}^{2}}{\sum_{i=1}^{n}{\left(P_{exp\left(i\right)}-{\bar{P}}_{exp\left(i\right)}\right)}^{2}}$$

### 3.5. Sensitivity Analysis

Finally, the sensitivity analysis was applied to find the level of impact of frequency, temperature, concentration as input variables in the modeling output variable can be found through the neural weight matrix. The equation required to carry out this analysis is known as the Garson equation based on the partitioning of connection weights:

(4) $$I_{j}=\frac{\sum_{m=1}^{m=N_{h}}\left(\frac{\left|W_{jm}^{ih}\right|}{\sum_{k=1}^{Ni}\left|w_{jm}^{ih}\right|}\times \;W_{mn}^{ho}\right)}{\sum_{m=1}^{k=N_{i}}\left\{\frac{W_{km}^{ih}}{\sum_{k=1}^{Nh}\left|w_{km}^{ih}\right|}\;\times \;W_{mn}^{ho}\right\}}$$

where *I_j_* is the relative importance of the frequency, temperature and concentration on the Z_re_, Z_im_ and Z_mod_, *N_i_* and *N_h_* are the quantity of input and hidden neurons, respectively; *W* are connection weights, the superscripts ”*I”*, “*h”* and “*o”* refer to input, hidden and output layers, respectively; and subscripts “*k”*, “*m”* and “*n”* refer to input, hidden and output neurons, respectively [35]. 

## 4. Results and Discussion

### 4.1. ANN Model

As mentioned earlier, an ANN training was used to predict the corrosion inhibition behavior for bronze in 3.5% M NaCl + 0.1 M Na_2_SO_4_ solution with the EIS database at 24 h of exposure to electrolyte; finding that the best architectures were Z_re_ (3:8:1), Z_im_ (3:16:1) and Z_mod_ (3:16:1) (see Figure 2) given that when the number of neurons is major to the values mentioned for each model, the coefficient *R*^2^ decreases and the *MSE* is major then the performance model was lower. All ANN models developed are described by the following equation:

(5) $$Z_{b}=\overset{S}{\underset{s=1}{\sum }}W_{o}\left(1,j\right)\times \;\left(\frac{2}{1+exp\left(-2\times \left(\sum_{k=1}^{K}(W_{i}\left(j,k\right)\times \;x_{i,Norm}\left(k\right))+b_{1}\left(j\right)\right)\right)}-1\right)+b_{2}$$

where *Z_b_* = Z_re_, Z_im_, and Z_mod_, *S* is the number of neurons in the hidden layer (*S* = 8, 16, 16), *k* is the number of neurons in the input layer (*K* = 3), *W* are weights and b the biases. The Table 2, Table 3 and Table 4 list the obtained parameters (*W_i_*, *W*_o_, *b*_1_, and *b*_2_) used for each ANN model; where *W_i_* represent weights in the hidden layer, *W*_0_ weights of the output layer; while *b*_1_ and *b*_2_ correspond to biases values in the hidden and output layer in the same order.

**Figure 3:**
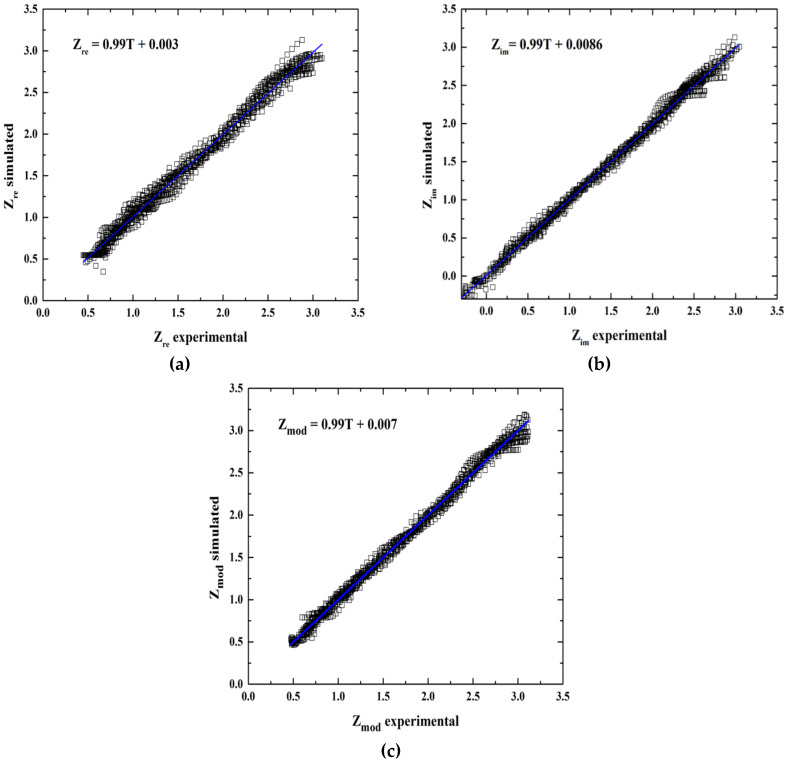
Regression performance of experimental and predicted values of electrochemical impedance spectroscopy for (**a**) Z_re_, (**b**) Z_im_ and (**c**) Z_mod_.

According to statistical analysis, the *R*^2^ value is reasonably high, which indicates the predictive power of the models (see Figure 3) for Z_re_ 0.9875, 0.9944 correspond to Z_im_, and finally 0.9876 for Z_mod_ (Table 5). In order to validate the ANN models, the intercept-slope test with 99% confidence was applied to demonstrate the linearity model, as mentioned before. The results are shown in Table 5, which indicates that the model is adequate to describe the behavior for inhibition corrosion of bronze considering that the slope = 1 and intercept = 0.

**Figure 4:**
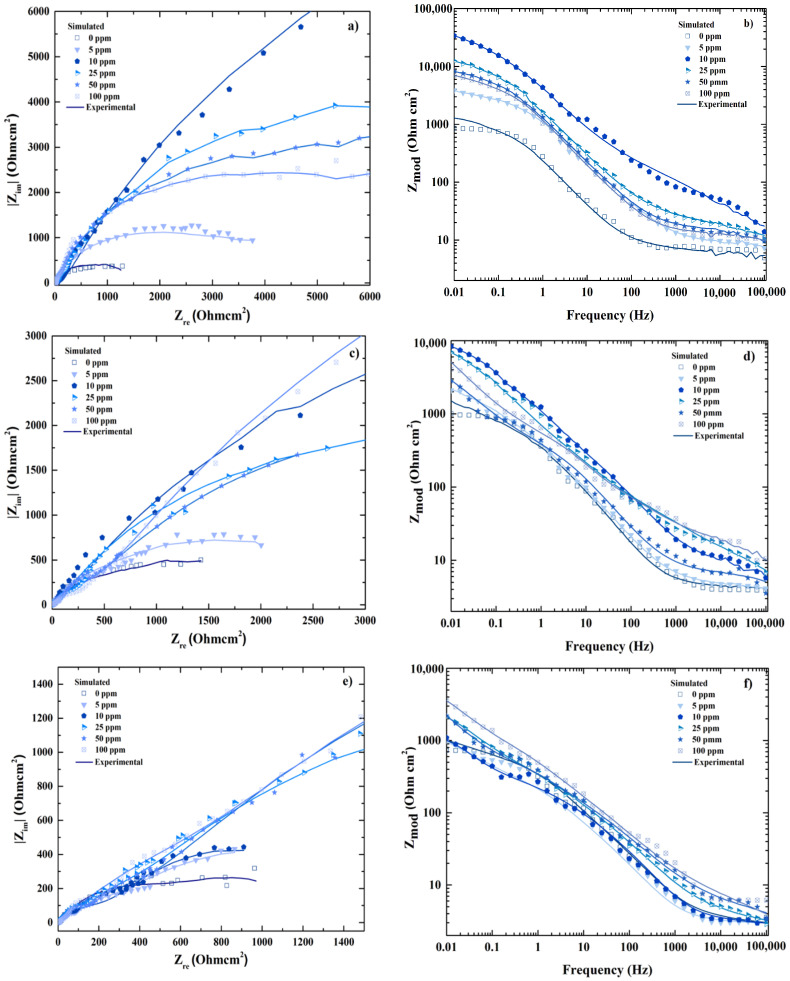
Comparison between experimental and simulated behavior of Electrochemical Impedance Spectroscopy (EIS) composed by Nyquist (**a**,**c**,**e**) and Bode spectrum (**b**,**d**,**f**) for 25, 40 and 60 °C respectively.

In addition, the comparison between the experimental and simulated results was possible plotting the spectrum EIS at the different temperatures (25, 40 and 60 °C) including inhibitor concentrations (0, 5, 10, 25, 50 and 100 ppm); the results obtained shows high correlation in Figure 4.

### 4.2. Sensitive Analysis of Input Variables

**Figure 5:**
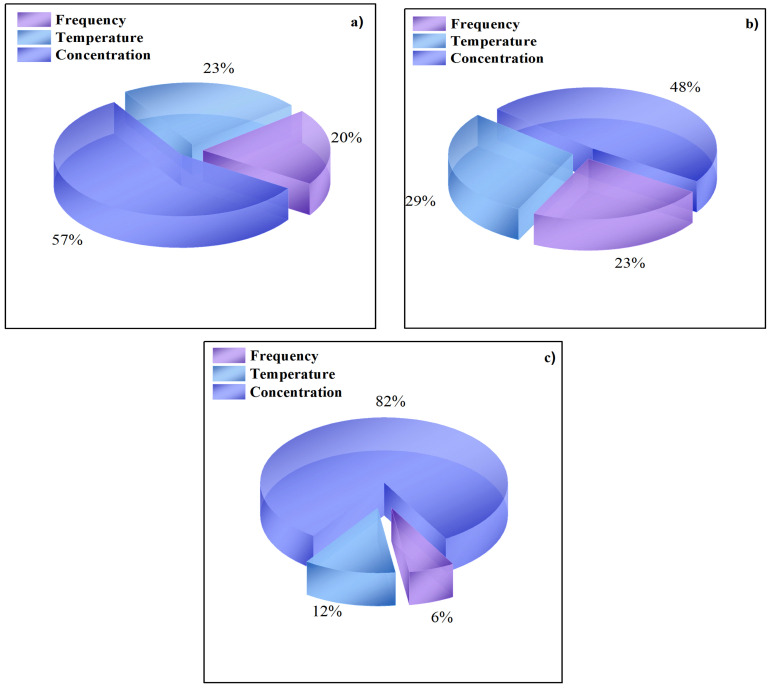
Relative importance (%) for input variables on the reponse to (**a**) Z_re_; (**b**) Z_im_ and (**c**) Z_mod_.

On the other hand, the sensitive analysis presented the same order of relative importance for the three ANN models. According to the results in Figure 5, the concentration represented the major relative importance followed by temperature and finally, the lowest percentage corresponded to the frequency in all cases; then the correct concentration measure could be considered as a critical parameter in the EIS test.

## 5. Conclusions

Three ANN models were developed and validated satisfactorily to describe the behavior for the inhibition corrosion of bronze in 3.5% NaCl + 0.1 M Na_2_SO_4_ indicating coefficients of determination equivalent to *R*^2^ = 0.9875, 0.9944, and 0.9876, for Z_re_, Z_im_, and Z_mod_ respectively. Additionally, the models achieved the intercept-slope test requirements.

The optimal architecture for Z_re_ model was obtained with (3:8:1) neurons, whereas for Z_im_ and Z_mod_ (3:16:1) neurons were used in the (input: hidden: output) layer respectively.

The sensitivity analysis revealed that, for the three ANN models, the variable with the greatest influence on the impedance response was the inhibitor concentration, followed by the temperature and the frequency.

Therefore, the three proposed ANN models can be used to estimate the variables involved in the EIS spectrum in a wide range of conditions extrapolating to other conditions of the same system of bronze/electrolyte.

## Figures and Tables

**Table 1:** Experimental intervals used to acquire the electrochemical impedance spectroscopy (EIS) values Input.

Variable		Interval	Units
Input	Frequency (f)	0.01–100,000	Hz
	Temperature (T)	25, 40 and 60	°C
	Concentration (X)	0, 5, 10, 25, 50 and 100	ppm
Output	Z_re_	2.43–30,730.92	Ω·cm^2^
	Z_im_	0.06–14,635.77	Ω·cm^2^
	Z_mod_	2.54–34,028.29	Ω·cm^2^

**Table 2:** Weights values (*W*_o_, *W_i_*) and biases (*b*_1_ and *b*_2_) for Z_re_ model (3:8:1).

Number of Neurons (*S*)	Weigths				Bias	
	Hidden Layer (*S* = 8, *K* = 3), *W_i_* = (*S*, *K*)			Output Layer (l = 1)	*b*_1_ (*S*)	*b*_2_ (l = 1)
	Temperature (*K* = 2)	Concentration (*K* = 3)	Frequency (*K* = 1)	*W_o_* (*S*)		
1	−33.646	−20.438	0.787	−2747.15	38.4	3852.324
2	−0.083	−0.218	−14,401.331	10,146.758	1440	-
3	−35.456	20.288	−0.783	2747.545	9.26	-
4	29.495	212.895	237.096	3870.101	−43.1	-
5	−0.084	−0.222	−14,575.934	−3403.786	1460	-
6	−3.519	20.429	−0.783	−2747.269	−4.93	-
7	0.285	−0.118	−184.512	978.928	14.4	-
8	−55.035	−20.405	0.783	−2747.447	10.8	-

**Table 3:** Weights values (*W_o_*, *W_i_*) and biases (*b*_1_ and *b*_2_) for Z_im_ model (3:16:1).

Number of Neurons (*S*)	Weigths				Bias	
	Hidden Layer (*S* = 16, *K* = 3), *W_i_*= (*S*, *K*)			Output Layer (l = 1)	*b*_1_ (*S*)	*b*_2_ (l = 1)
	Temperature (*K* = 2)	Concentration (*K* = 3)	Frequency (*K* = 1)	*W_o_* (*S*)		
1	6.614	−23.974	0	−764.894	−3.78	650.218
2	−0.036	−0.011	9166.232	−1805.728	−915	-
3	−0.191	0.322	21.357	−102.331	0.665	-
4	−332.336	−192.892	0.786	−363.086	329	-
5	−135.744	−142.424	0.003	650.751	142	-
6	1302.857	1486.882	361.305	368.812	−1950	-
7	−1.379	−4.616	−188.514	247.849	16.9	-
8	236.332	−946.128	93.239	97.318	173	-
9	11.089	−9.867	−0.003	−585.071	0.621	-
10	6.134	−3.169	−0.371	−21.576	0.216	-
11	−14.635	−17.402	−0.018	−291.533	10.9	-
12	−0.316	0.135	−262.205	187.906	23.3	-
13	−0.042	−0.012	9380.565	629.753	−937	-
14	−4.066	2.754	0.02	−396.08	−0.54	-
15	5.255	−17.41	−0.015	287.948	2.15	-
16	1.68	3.64	169.097	510.83	−14.6	-

**Table 4:** Weights values (*W_o_*, *W_i_*) and biases (*b*_1_ and *b*_2_) for Z_mod_ model (3:16:1).

Number of Neurons (*S*)	Weigths				Bias	
	Hidden Layer (*S* = 16, *K* = 3), *W_i_* = (*S*, *K*)			Output Layer (l = 1)	*b*_1_ (*S*)	*b*_2_ (l = 1)
	Temperature (*K* = 2)	Concentration (*K* = 3)	Frequency (*K* = 1)	*W_o_* (*S*)		
1	−33.176	17.960	0.012	−547.000	−17.189	552.201
2	3.805	−69.199	1271.784	527.000	−61.965	-
3	0.083	1.466	251.299	−107.000	−22.409	-
4	−0.018	−0.092	−6863.647	−1800.000	685.730	-
5	−0.944	25.642	−0.678	−235.000	−7.706	-
6	−22.081	−11.516	0.534	−49.100	23.759	-
7	−0.017	−0.100	−7271.011	654.000	726.734	-
8	−33.898	−11.118	0.529	49.200	34.268	-
9	−0.940	21.966	−0.679	236.000	−6.602	-
10	−0.601	−0.365	37.056	−168.000	0.104	-
11	4.029	145.354	114.095	547.000	−22.550	-
12	−17.393	−10.582	15.370	0.084	8.632	-
13	20.647	−2.031	1124.730	−117.000	−110.286	-
14	−272.457	−176.891	278.738	−0.078	123.102	-
15	−0.020	−0.083	−6356.422	2930.000	634.291	-
16	−1982.569	417.592	−660.569	0.112	735.831	-

**Table 5:** Results of statistical analysis with intercept-slope test.

Output Variable	Architecture	*R* ^2^	*MSE*	Intercept Slope Test			
				a_min_	a_max_	b_max_	b_min_
Z_re_	3:8:1	0.9875	0.00659	0.0251	−0.0079	1.0049	0.9862
Z_im_	3:16:1	0.9944	0.00475	0.0186	−0.0013	1.0002	0.9878
Z_mod_	3:16:1	0.9876	0.00686	0.0198	−0.0059	1.0009	0.9873
